# Exploring the Loss Aversion Scale’s psychometric properties in Spain

**DOI:** 10.1038/s41598-024-66695-6

**Published:** 2024-07-08

**Authors:** Javier Cabedo-Peris, César Merino-Soto, Guillermo M. Chans, Manuel Martí-Vilar

**Affiliations:** 1 Department of Basic Psychology, Faculty of Psychology and Speech Therapy, Universitat de València, Valencia, Spain; 2 Institute for the Future of Education, Tecnologico de Monterrey, Monterrey, Mexico; 3 Institute for Research in Psychology, University of San Martín de Porres, Lima, Peru; 4 School of Engineering and Sciences, Tecnologico de Monterrey, Mexico City, Mexico

**Keywords:** Loss aversion, Higher education, Risk behavior, Validity, Social desirability, Educational innovation, Psychology, Human behaviour

## Abstract

Loss aversion is a psychological construct defined as a tendency to value potential losses more than gains in a situation that requires decision-making. The Loss Aversion Scale (LAS, eight items) measures an individual’s loss aversion to various situations. However, the generalization of its psychometric properties to different population groups is unknown. This study aimed to validate the LAS instrument for use among Spanish university adults. To this end, two studies were conducted: a content validity study calculating the substantive validity (N = 24) of the instrument’s translation from original English to Spanish and a study of internal structure and association (N = 766) among Spanish university men and women aged 18–35. The analyses performed for each sample indicated that the instrument had adequate validity and reliability values as a one-dimensional measure; however, items 5 and 8 had to be removed. Their scores indicated moderate-magnitude correlations with social desirability. This article debates the study’s limitations, practical implications, and future lines of research based on the results. The conclusion is that the Loss Aversion Scale instrument suits general Spanish population samples and requires probable methodological control concerning social desirability.

## Introduction

### Loss Aversion

The concept of “loss aversion” (LA) finds its roots in prospect theory, introduced by Kahneman and Tversky in 1979^1^. Prospect theory aims to explain decision-making in economics, which differs from the traditional expected utility model^2,3^ and the subjective utility theory^4^. Prospect theory, initially studied from an economic perspective, made its foray into health and psychology in 1987. Meyerowitz and Chaiken discovered that individuals who received information emphasizing loss were more inclined to perform breast self-examinations compared to those receiving information emphasizing benefits^5^. Recent topics reviewed include behavioral addiction^6^, immunotherapy^7^, and hearing aids^8^.

Prospect theory clearly distinguishes between risk aversion and LA. Risk aversion is the tendency to choose certainty over uncertainty or risk^9^. On the other hand, LA is a subjective perception where losses loom larger than equivalent gains from a reference point^1,10,11^. This study focuses specifically on LA.

LA has been discussed from two different perspectives. First, it has been considered a global phenomenon affecting the general population, where potential losses are always deemed more painful than the happiness derived from equivalent potential gains^1,11^. Secondly, recent findings suggest this is not always the case. Specific scenarios exist where potential gains can have the same or even greater impact on people’s perceptions^12,13^. This situation can depend on factors such as the magnitude of the loss^14,15^ or potential moderators not previously studied^12,16^. Due to this variations, the LA effect has even been considered a “fallacy” ^17^.

However, some studies have sought to refute claims that LA is not a real phenomenon^18,19^. They suggest that moderators influence how a person is affected by LA, including the methods used to measure it or sociodemographic variables. These studies employed different techniques and measured various sociodemographic factors to support the existence of LA. Evidence shows that LA persists across both risky and riskless choices^19,20^ and is prevalent across populations regardless of income, perceived gains or losses, and whether decisions revert individuals to their previous status quo^18^.

Regarding sociodemographic variables, being older and wealthier tends to increase LA, while having a higher educational level tends to decrease it^18,19^. These findings are consistent with other recent studies measuring LA through various moderators, such as age^21^, culture^22^ or neurophysiology^23^. Collectively, these points demonstrate that LA exists and can be measured at an individual level. The present study aligns with this assertion.

LA has been linked to various topics that have societal implications. Review articles on LA in health indicate its examination in human social decision-making, prosocial behaviors^24^, biological medicine^25^, diabetes^26^, and substance and behavioral addictions^27^. According to the latter article, consensus on how to measure LA remains elusive. Some of the measurement tools that appear in that review are directed to measure other variables different from LA, such as impulsiveness^28^, decision-making^29^, time preferences^30^, risk aversion^30^, among others. The review underscores the need for a unified instrument validated across various countries to ensure the generalizability of results.

### Loss aversion measurement

According to the definition of LA, it depends on a specific reference point^1,10^. The lambda parameter (*λ*) is typically used in its measurement. Preferences for choosing various options are evaluated based on this reference point^11^. By its nature, the utility value of an outcome (*U*) depends solely on the outcome itself. However, according to prospect theory, *U* must also consider the parameter *λ*. Following what prospect theory states about LA, *U* is steeper for losses than for gains^31^. Scientific literature identifies several paradigms that evidence LA^11–14^. Some of the most popular include the status quo bias^32^, the endowment effect^33^, the sunk cost fallacy^34^, the risky bet premium^10^, and the framing effect^9^.

Most studies use choice tasks with different levels of risk to verify LA across their data. In particular, willingness-to-accept or willingness-to-pay tasks have been commonly used^35^. Willingness-to-accept tasks measure the smallest amount of money someone would accept in exchange for selling something, while willingness-to-pay tasks measure the highest amount someone would be willing to pay for something^36^. Following the endowment effect, the difference between willingness-to-accept and willingness-to-pay tasks can be considered as a proof of LA^37^.

Exercises based on lotteries are usually used to measure LA through risky choice tasks^38,39^. However, these choice-task experiments can be time-consuming and complex. Additionally, they may be biased by the description and particularities of the choices, which hinders generalization.

In 2021, the Loss Aversion Scale (LAS) emerged to measure LA^13^. The LAS consists of eight items designed to assess individuals’ perceptions of losses and gains, failures and successes, and the duration of these emotional experiences. During its development, the LAS demonstrated convergent and divergent validity with similar constructs such as risk aversion and risk propensity. Notably, the LAS offers advantages over other instruments, including ease of implementation as a self-reporting questionnaire that does not require experimental choice tests, thus streamlining the assessment process while targeting the analysis of the LA variable. Also, it is supposed to overcome the limitations reported before by presenting items general enough to be adapted to any specific scenario.

On the contrary, the wording of the LAS items lends itself well to adaptation for use in various contexts, allowing for modifications in the content of instructions. Its self-reporting format easily integrates into other self-reporting assessments, facilitating analysis through routine quantitative techniques for validation or correlation with different variables.

Historically regarded as a cognitive bias, LA likely manifests as a response tendency influenced by individual characteristics such as personality and gender, with studies indicating higher levels in women^40^. Moreover, emerging research suggests that the brain’s amygdala modulates the noradrenergic pathway while evaluating potential losses, contributing to heightened levels of LA^41^. Consequently, this construct does not appear confined to a specific cultural context, imparting emic significance to the LAS instrument.

The recent development of the LAS may partially account for the limited number of validation studies conducted thus far; only one such study has been reported, focusing on the cultural context of Nigeria^42^. This study revealed certain shortcomings of the instrument when used among young Nigerian entrepreneurs. The LAS, comprising eight items and demonstrating a dimensionality of three factors as determined by Exploratory Factor Analysis, exhibited a reliability coefficient of *α* = 0.72. However, the study did not include an analysis of measurement invariance, identification of careless responses, or item analyses. Consequently, the present study represents the first validation of the LAS instrument in a language other than English, offering an in-depth examination of its properties.

### Study of the properties of the scale

The adaptation and examination of the properties of the LAS instrument in languages other than English are deemed essential for its broader applicability and generalizability across diverse populations. To ensure standardized validation procedures, the Standards for Educational and Psychological Testing^43^ offer a comprehensive framework for guiding the validation process. According to these guidelines, validation necessitates accumulating empirical evidence from various sources to establish validity.

In the current study, conducted in Spain, the validation of the LAS instrument relied on multiple sources of validity, including content validity, internal structure, and correlation with other variables. By adhering to these established standards, researchers can effectively assess the suitability and reliability of the LAS instrument for use in different linguistic and cultural contexts.

### The current study

Research Question: How can the LAS instrument be used among young Spanish adults?

Overall Objective: Regarding the gaps in the abovementioned review^27^, this study aimed to validate the LAS^13^ in a Spanish sample.

Specific Objectives:

Specific Objective 1. Obtain evidence of content validity.

To thoroughly examine the evidence, we performed a quantitative item analysis to assess the content’s validity^44^ and determine its substantive validity. In the present study, the substantive validity assessed concerns the measurement structure of the LAS, not the underlying processes linked to the construct^45^. Substantive validity refers to the relationship between the content (items) and the meaning of the construct, as judged by the respondents^46,47^.

Specific Objective 2. Analyze the validity of the internal structure.

Determining the validity of the internal structure consisted of examining dimensionality, measurement invariance, and reliability.

Specific Objective 3. Explore the validity of correlation with other variables.

The validity of the correlation with other variables came from exploring the relationship of LAS scores with psychological dependence on smoking and sensation seeking (as divergent variables) and social desirability (to detect possible biases). The selection of these variables is based on several considerations. First, the relationship with psychological dependence on tobacco is supported by evidence suggesting a correlation between addictive behaviors and LA. Specifically, individuals who use drugs tend to exhibit lower LA^48–50^. Recent literature shows that cigarette smokers have lower LA compared to non-smokers^51–53^.

Secondly, the correlation with sensation-seeking was examined to assess an anticipated negative relationship between these variables, consistent with findings from other recent psychology studies^54,55^. This rationale is grounded in the definition of LA itself.

Lastly, the relationship with social desirability was investigated to identify potential biases, particularly when studying behaviors influenced by social approval^56^, even in online questionnaires^57^.

However, more research is needed to confirm that these variables are strictly correlated. Therefore, this study aims to explore how LA may correlate with other variables through exploratory analyses.

Four hypotheses were constructed within the framework of the expected validity evidence.

Hypothesis 1. When translated into Spanish, the LAS will present adequate validity and reliability levels to be considered suitable for Spanish samples.

Hypothesis 2. A moderately negative correlation is hypothesized between psychological dependence on tobacco, as measured by the Glover-Nilsson Test scale^58^, and tobacco use over the past year. Individuals with high LA are expected to avoid tobacco use due to its potential losses and negative consequences.

Hypothesis 3. A high negative correlation is expected between LAS and sensation seeking, as measured by the Brief Sensation Seeking Scale instrument^59^. Based on the previous arguments, individuals with high LA are assumed to avoid sensation-seeking behaviors.

Hypothesis 4. There will be no association (i.e., zero correlation) between LAS and social desirability, as measured by the Social Desirability Scale^60^.

## Methods

### Study design and participants

The current study follows a cross-sectional design. The initial reference population consisted of second and third-year university students enrolled in the Bachelor’s Degree in Psychology program at the University of Valencia (Valencia, Spain). However, the sample was expanded to include a broader population by encouraging participants to share the questionnaire with others. The sample was incidental. All the students meeting the inclusion criteria of age (over 18 and under 35) and nationality (Spanish) were asked to share the questionnaire with their relatives. The population of the present study comprised two non-probabilistic convenience samples.

**Sample 1.** For the LAS study of substantive validity, a component of content validity, the sample comprised 24 Spanish university students (50% women) pursuing different degrees. The mean age was 26.3 (SD = 5.08).

**Sample 2.** This sample passed through the analysis of the second component of content validity (i.e., item analysis), internal structure, and correlation with other variables. There were 837 subjects initially, but data were excluded for the reasons below. Aware that cultural differences could influence the study, the authors restricted the sample to the Spanish population only, reducing the total to 792. The authors consider it essential to conduct cross-cultural studies to observe possible differences in the variables. Finally, 26 insufficient or careless responses from participants were eliminated (see the Data Quality subsection). The final sample totaled 766 subjects (62.09% women). The mean age was 23.67 (SD = 4.19). Most of the population resided in the Valencian Community (75.6%); the predominant level of study was university (46.7%). Table 1 shows the sociodemographic categories in Sample 2.

**Table 1:** Sociodemographic characteristics of the sample.

Level of studies	Frequency	%	Region in Spain	Frequency	%	Tobacco use during the past year	Frequency	%
SE	1	0.1	CV	579	75.6	Never	244	31.9
EP	11	1.4	CM	29	3.8	Once a year	100	13.1
ES	29	3.8	A	25	3.2	Once a month	37	4.8
B	136	17.8	CLM	21	2.7	Once per	77	10
FPM	30	3.9	C	17	2.2	Week		
FPS	85	11.1	AR	12	1.6	Daily	308	40.2
GU	358	46.7	PV	12	1.6			
MU	111	14.5	Other	42	5.5			
D	5	0.7	NR	29	3.8			
Total	766	100		766	100		766	100

Note. SE: no studies. EP: primary education. ES: secondary education. B: High school. FPM: intermediate vocational training. FPS: higher level vocational training. GU: Undergraduate Degree. MU: Master’s Degree. D: Doctorate. CV: Community of Valencia. CM: Community of Madrid. A: Andalucía. CLM: Castilla-La Mancha. C: Cataluña. AR: Aragón. PV: Basque Country. Other: Autonomous Communities with less than 10 people represented. NR: No response.

In the final sample, gender differences were found in final-year students using tobacco (biserial point *r* =  − 0.142; CI95% =  − 0.207, − 0.072; Wilcoxon’s test = 444,456; *p* < 0.001), age (biserial point *r* =  − 0.116; CI95% =  − 0.185, − 0.046; Wilcoxon’ test = 586,756; *p* < 0.001), and the level of education attained (biserial point *r* = 0.071; CI95% = 4.45e − 06, 0.146; Wilcoxon’s test = 584,669; *p* < 0.001). However, a recent review of 134 meta-analyses found these results minor^61^. The variables of age and level of education were below the 25th percentile (*r* = 0.12), and tobacco consumption was slightly above.

### Instruments

**Loss Aversion Scale (LAS)**^13^. This self-reporting assessment measuring individual LA in different scenarios consists of eight items (e.g., “I feel nervous when I have to make a decision that may lead to loss”). It was double-translated into Spanish by English language experts. It is scored on a 5-point Likert scale ranging from 1 = “completely disagree” to 5 = “completely agree”. The LAS score is calculated by summing the responses to all items, except for items 5 and 8, which are reverse-coded and must be inverted before inclusion. High scores indicate greater LA. The reliability of the LAS is generally moderate^13^.

**Perceived Clarity of the Items.** Participants were presented with the eight LAS items without disclosing the instrument they belonged to and were asked to rate each item on a scale from 1 (not at all clear or understandable) to 5 (completely clear or understandable).

**Substantive validity.** Students were provided with descriptions of four concepts: loss aversion, sensation seeking, empathy, and social desirability. Loss aversion was described as valuing the negative consequences of a behavior over the positive ones. Sensation seeking was defined as the need to seek and experience new, varied, complex, and intense experiences and sensations, along with the desire to take physical and social risks to enjoy such experiences. Empathy was defined as the ability to put oneself in another person's shoes and understand their emotions, reactions, feelings, and behaviors, even to the point of connecting with them. Finally, social desirability was described as the desire to project a socially appropriate or acceptable image of oneself. After reading these definitions, participants were again presented with the eight LAS items and asked to assign each item to the corresponding concept: 1 = sensation seeking, 2 = loss aversion, 3 = social desirability, and 4 = empathy.

**Glover-Nilsson Test**^58^. It measures psychological and behavioral dependence on tobacco at present without considering purely physiological dependence. The Spanish version (11 items)^62^ was used (e.g., “Do you place something in your mouth to distract you from smoking?”). The ordinal scale responses ranged from 1 = “nothing at all” to 5 = “extremely.” Participants had been previously asked if they had smoked tobacco during the last year. They were asked to choose between “never,” “more than once a year,” “more than once a month,” “more than once a week,” and “daily.” Those who answered “never” were excluded from the Glover-Nilsson Test. The McDonald’s *ω* coefficient of the questionnaire in this study was high (*ω* = 0.923, CI95% = 0.911, 0.939; *α* = 0.888, CI95% = 0.873, 0.9).

**Brief Sensation Seeking Scale**^59^. It measures the dimension of temperament disposed to search for new experiences that can bring exciting sensations (e.g., “I would like to explore strange places”). The 4-item version was used^59^. Hispanic validation studies of this version have shown satisfactory psychometric properties^63,64^. The Likert-scale responses ranged from 1 = “completely disagree” to 5 = “completely agree”. This study’s reliability was acceptable (*ω* = 0.677, CI95% = 0.639, 0.714; *α* = 0.659, CI95% = 0.616, 0.698).

**Social Desirability Scale**^60^. It measures the tendency to select the socially acceptable response. It comprises 11 items in Spanish (e.g., “*A la mayoría de las personas les gusta a chismear a veces*”: Most people like to gossip sometimes). High scores are interpreted as a stronger tendency to follow socially desirable behavior. The five-option Likert scale responses ranged from 1 = “completely disagree” to 5 = “completely agree.” This study’s reliability was high (*ω* = 0.907, CI95% = 0.892, 0.924; *α* = 0.872, CI95% = 0.854, 0.887).

### Procedure

**Language adaptation (translation).** The LAS was initially developed and validated in English. To use it in the present study, we employed a back-translation process^65^. First, a native Spanish speaker with advanced proficiency in Cambridge English translated the scale into Spanish. Subsequently, a bilingual expert, fluent in Spanish and English and possessing expertise in psychology, conducted a second translation back into English. The authors meticulously compared the back-translated version with the original English scale, revealing no significant disparities, which indicated a high degree of equivalence between the original and double-translated scales.

Given that the sample was drawn from Spain, the translated material was adjusted to reflect regional Spanish variations while excluding colloquialisms exclusive to specific regional dialects. This approach ensured that the Spanish version maintained broad applicability across various Spanish-speaking regions. Both expert translators prioritized standard, easily comprehensible vocabulary accessible to Spanish speakers in Spain and across Hispanic countries.

**Data collection.** Participants were recruited in person. Researchers visited the Faculty of Psychology and Speech Therapy of the Universitat de València (Valencia, Spain) and requested permission from professors to enter their classrooms. The researchers provided students with a link to access the questionnaires hosted on LimeSurvey. Participation was voluntary, and those who chose to participate were asked to complete the questionnaires immediately. They could use their personal computers or any electronic device with internet access. Researchers remained in the classroom to assist with any questions until the last participant finished.

Data collection was organized according to the sample. First, participants were selected from sample 1 to assess the perceived clarity of the items. A group of 12 girls and 12 boys was chosen, ensuring they fell within the specified age range (18 to 35 years). Most participants came from the same classrooms initially accessed, and those who did not meet the age criteria were subsequently selected through student contacts. Socio-demographic data were collected, including age, biological sex, highest level of education attained, and nationality.

For the collection of data related to content validity, internal structure, and relationships with other variables, sample 2 was used. The initial segment of the questionnaire included sociodemographic inquiries, prompting participants to disclose details such as age, gender, current education status, nationality, region in Spain in which they are currently living, and tobacco use within the past year. Subsequently, participants addressed queries related to LA, psychological tobacco dependency, sensation seeking, and social desirability. After completing the questionnaires, sample 2 participants were encouraged to share the link with their relatives who met the inclusion criteria for age and nationality, facilitating a snowball recruitment procedure.

All participants volunteered for the study by reading and accepting the informed consent form. They were assured of their freedom to withdraw from the experiment at any moment without repercussions. Upon completing the questionnaires, participants received a thank-you message from the researchers but no further compensation.

No missing data were found because the survey was configured to prevent progression to the next section until the current section was fully completed. On average, sample 2 participants took 451.13 s (7.5 min) to complete the questionnaires, with a standard deviation of 416 s (6.9 min). Data collection was conducted online and lasted from June to December 2023.

### Data analysis

**Figure 1:**
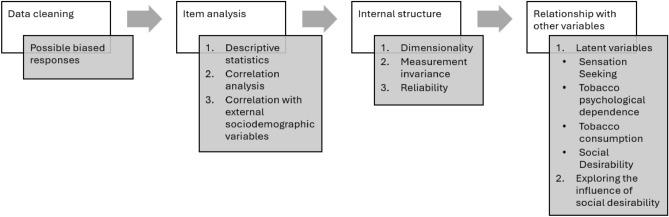
Order of execution of the analyses carried out on Sample 2.

The analyses performed on Sample 2 of the study are presented in the sequence depicted in Fig. 1.

**Possible biased responses.** First, methods were implemented to evaluate and ensure the quality of the data collected in Sample 2, aiming to reduce potential Type I or Type II errors^66^. After filtering participants by gender and nationality (see Participants section), responses exhibiting insufficient effort or carelessness were identified. These occur when items are not answered attentively^67^, and can manifest as excessive response consistency (e.g., identical consecutive responses) or inconsistency (e.g., responses that deviate significantly from the reference group’s common pattern)^67,68^. Non-parametric methods were used to detect responses with insufficient effort or carelessness: “G_+_”^69^ and “O_+_”^68^. These methods detect excessively inconsistent and consistent responses in categorical variables, respectively. In both methods, the count of Guttman errors and infrequent scores is obtained for each respondent, and the distribution of each is included with a cut-off point established by Tukey fences (3rd quartile + 1.5 × Interquartile range)^68^. G_+_ and O_+_ scores beyond the cut-off point were removed.

**Validity of content.** In Sample 1 (n = 24), the perceived item clarity was evaluated, quantifying it with the mean clarity for each item with asymmetric confidence intervals^70^. In addition, substantive validity^46^ was calculated using the coefficients of substantive agreement and substantive validity. The sample size in both procedures was sufficient for a pretest^46,71^. Finally, the analysis of items in Sample 2 (n = 766) reported the descriptive statistics; subsequently, the correlations between them were examined. Content validity was calculated using the R *rcompanion package* for the Spearman rho test^72^. In this study, the response trend of each LAS item and its construct validity were determined for the variables of sex, age, level of education attained, and tobacco consumption during the past year.

**Internal structure.** Three specific pieces of evidence were obtained for Sample 2: dimensionality, measurement invariance, and reliability.

*Dimensionality.* Confirmatory Factor Analysis was performed, treating the items as ordinal categorical variables and estimating inter-item polychoric correlations. First, a one-dimensional model that included the original eight instrument items was tested. The Unweighted Least Square Mean Variance estimator was used^73^. The statistical test was the goodness of fit *χ*^2^, supplemented by approximate adjustment indices. The adjustment of fit indices to assess how the models fit the data were the Comparative Fit Index (CFI > 0.95), the Root Mean Square Error of Approximation (RMSEA < 0.05) and the Standardized Root Mean Square Residual (SRMR < 0.05).

*Measurement invariance.* The measurement invariance analysis of the LAS instrument encompassed variables such as gender, age, and tobacco consumption within the past year. Gender categorization distinguished between men and women, while age was stratified into two groups: youths (18–27 years old) and young adults (28–35 years old). Tobacco use was classified as either light (those who reported never using or abstaining in the past year) or habitual (respondents who admitted to using at least once per month, week, or day).

Following the framework of dimensionality analysis (utilizing the same estimator, categorical variables, approximate adjustment indices), we evaluated the measurement invariance, incorporating Wu and Estabrook^74^ specifications to assess measurement invariance in categorical variables. This process involved imposing successive equality constraints, as outlined by Muthén and Asparouhov^75^. The sequence comprised configurational invariance, wherein the number of dimensions was set as equal; metric invariance, where factor loads and thresholds were constrained equally; and scalar invariance, entailing equal intercepts.

The equality of the invariance models was tested with the difference in the statistical test of each model (Δ*χ*^2^) and in the approximate adjustment indices, with the following criteria: ΔCFI ≤ 0.01, ΔRMSEA < 0.01, ΔSRMR < 0.015^76^.

The evaluation of measurement equivalence was completed with a non-equivalence size indicator using the coefficient d_MACS_^77^. This metric, interpreted as the standardized difference *d*^78^, or the degree of bias or non-equivalence in item response due to the absence of metric and/or scalar invariance, and in the total score, offered categorization into trivial (< 0.40), low (≥ 0.40), medium (≥ 0.60), and high (≥ 0.80) levels of non-equivalence^79^.

*Reliability*. The reliability of the score was calculated with the omega coefficient. For comparison with other studies, we also calculated the alpha coefficient. Practical value level indicators were assessed using the standard error of measurement.

**Relationship with other variables.** The association between LAS and external variables was made by evaluating the correlations between variables measured for Sample 2, using structural equation modeling, and defining the variables as latent. Following the definition of LA, we chose variables to explore divergence between them, such as sensation seeking, using the Brief Sensation Seeking Scale^59^, and psychological dependence on tobacco, as measured by the Glover-Nilsson Test^58^ and tobacco use in the past year. In addition, the absence of social desirability biases was assessed using the Social Desirability Scale^60^. We performed regression and covariance analyses on the latent variables to evaluate the possible influence of social desirability on the other variables.

The analyses were performed using the R packages: *rcompanion*^72^, *sjmisc*^80^, *DescTools*^81^, *MVN*^82^, *lavaan*^83^, *semTools*^84^, and *mokken*^85^.

### Ethics approval and consent to participate

The Human Research Ethics Committee of the Experimental Research Ethics Committee at the University of Valencia (Valencia, Spain) supported this study. This body evaluated and approved the procedure and the instruments used, reporting it favorably under the code 2023-MAG-3174468.

This research was aligned with the principles of the Declaration of Helsinki^86^ and the Belmont Report^87^. All procedures were conducted in strict accordance with the applicable guidelines and regulations. All participants, without exception, consented to their participation on the condition of total anonymity.

## Results

### Content validity

**Language adaptation (translation).** Table 2 shows the LAS translated into Spanish. The back-translation was reviewed by both translators, who reached a consensus. The authors of this study found no significant differences, meaning that the original scale and the double-translated scale were highly similar. There was only one concern regarding the word “encourages” in item 7. It was agreed to translate it as “motiva” (*motivates*) instead of “anima” or “alienta” (*encourages*) because the item implied a directionality, and it was better represented by “motiva” in Spanish. The authors assessed the adapted instrument as linguistically and semantically appropriate.

**Table 2:** LAS translated into Spanish and in its original version.

Escala de Aversión a la Pérdida *Loss Aversion Scale*
1. Cuando tomo una decisión, pienso mucho más en lo que perdería que en lo que ganaría *When making a decision, I think much more about what might be lost than what might be gained*
2. El dolor de perder dinero tiene más importancia que el placer de ganar la misma cantidad de dinero *The pain of losing money matters more than the pleasure of gaining the same amount of money*
3. Me pongo nervioso cuando tengo que tomar una decisión que puede llevar a la pérdida *I feel nervous when I have to make a decision that may lead to loss*
4. Para mí, el dolor de perder algo tiene más importancia que el placer de conseguirlo *The pain from losing something matters much more to me than the pleasure from getting it*
5. Para mí, evitar el fallo es menos importante que perseguir el éxito (codificación inversa) *Avoiding failure is less important to me than seeking success (reverse coding)*
6. Experimentar una gran pérdida permanece más en la mente que experimentar una gran ganancia *Experiencing a major loss stays in my mind longer than experiencing a major gain*
7. Me da más miedo un posible fallo de lo que me motiva una posible ganancia *A potential failure scares me more than a potential success encourages me*
8. El sufrimiento que viene con las pérdidas puede ser compensado totalmente por el placer que viene con las ganancias (codificación inversa) *The suffering that comes with losses can be fully offset by the pleasure that comes from gains (reverse coding)*

**Clarity of items.** The perceived clarity of the items received high average scores in Sample 1 (> 3.90 in each item; Table 3). The upper limit of the calculated interval reached values greater than 4.2. These results indicate that the clarity of the items was satisfactory in the sample.

**Table 3:** Clarity and substantive validity of LAS.

	M	90% confidence interval		Observations
		LL	UL	
LAS_1	4.750	4.400	5.000	None
LAS_2	4.250	3.879	4.621	None
LAS_3	4.417	4.050	4.783	None
LAS_4	4.708	4.356	5.000	None
LAS_5	3.958	3.583	4.334	None
LAS_6	4.667	4.312	5.000	None
LAS_7	4.500	4.137	4.863	None
LAS_8	4.125	3.752	4.498	None

Note. LAS_1 … LAS_8: Loss Aversion Scale items. M: response mean. LL: lower limit. UL: upper limit. Observations: comments from the sample about the content of the items.

**Substantive validity.** The coefficients of substantive agreement were high for Sample 1, except low for items 5 and 8 (Table 4). All substantive validity coefficients were high (> 0.50), except items 5 and 8, which showed negative coefficients, indicating that perceived conceptual substantiveness was strong in other constructs. Table 4 shows that the sensation-seeking construct seems to overlap with both items, while the social desirability and empathy constructs were distinctive concerning LA. Items that did not fit their constructs were identified as content that was possibly not representative of them and were recognized as objectives in the dimensionality assessment.

**Table 4:** Assessment of the substantive validity of LAS items.

	Sensation seeking (SS)	Loss aversion (LA)	Social desirability (SD)	Empathy (Emp)	*c*_*sv*_
LAS_1					0.667
Freq.	3	20	0	1	
Prop.	0.00	0.952	0.00	0.048	
90% CI	0.00, 0.144	0.812, 0.989	0.00, 0.011	0.011, 0.188	
LAS_2					0.917
Freq.	0	23	1	0	
Prop.	0.00	0.958	0.042	0.00	
90% CI	0.00, 0.101	0.833, 0.991	0.009, 0.167	0.00, 0.101	
LAS_3					0.833
Freq.	1	22	1	0	
Prop.	0.042	0.917	0.042	0.00	
90% CI	0.099, 0.167	0.777, 0.972	0.009, 0.167	0.00, 0.101	
LAS_4					0.917
Freq.	0	23	1	0	
Prop.	0.00	0.952	0.048	0.00	
90% CI	0.00, 0.114	0.812, 0.989	0.011, 0.188	0.00, 0.114	
LAS_5					− 0.333
Freq.	13	8	3	0	
Prop.	0.542	0.333	0.125	0.000	
90% CI	0.379, 0.696	0.199, 0.501	0.051, 0.275	0.00, 0.101	
LAS_6					0.833
Freq.	1	22	0	1	
Prop.	0.042	0.917	0.00	0.042	
90% CI	0.099, 0.167	0.777, 0.972	0.00, 0.101	0.099, 0.167	
LAS_7					0.917
Freq.	0	23	0	1	
Prop.	0.00	0.852	0.00	0.148	
90% CI	0.00, 0.091	0.708, 0.932	0.00, 0.091	0.068, 0.292	
LAS_8					− 0.333
Freq.	14	8	1	1	
Prop.	0.583	0.333	0.042	0.042	
90% CI	0.418, 0.732	0.199, 0.501	0.009, 0.167	0.009, 0.167	

Note. LAS_1 … LAS_8: Loss Aversion Scale items. Freq: response frequency. Prop: response proportion. CI: confidence interval. SS: sensation seeking. LA: loss Aversion. SD: social desirability. Emp: empathy. *c*_*sv*_: substantive validity coefficient.

**Item analysis.** The inter-item polychoric correlations for Sample 2 had a magnitude that can be considered high in this context of categorical variable analysis (Table 5). Items 5 and 8, however, showed extremely low correlations around zero (item 5, M = 0.11, Min = 0.03, Max = 0.16; item 8, M = 0.03, Min =  − 0.08, Max = 0.16). This magnitude of correlations does not contribute to the dimensionality of the LAS and, therefore, to its conceptual unity. These items had previously been flagged as problematic during substantive validity assessment. Items 5 and 8 were formulated in negative, indicating that they should be reworded. However, only a negative correlation between items 8 and 4 was observed, with the rest being low but positive. None of the correlations were considered problematic because they had a high association.

**Table 5:** Statistics and correlations for the Loss Aversion Scale (LAS) items.

Item	Descriptions					Inter-item correlations^a^							
	M	SD	Sk	Ku	AD	LAS_1	LAS_2	LAS_3	LAS_4	LAS_5	LAS_6	LAS_7	LAS_8
LAS_1	3.39	1.15	− 0.37	− 0.75	3.03	1							
LAS_2	3.28	1.21	− 0.29	− 0.9	27.81	0.36	1						
LAS_3	4.04	0.99	− 1.12	0.86	56.38	0.46	0.37	1					
LAS_4	3.23	1.21	− 0.24	− 0.95	28.13	0.46	0.52	0.48	1				
LAS_5	3.07	1.18	− 0.09	− 0.97	27.15	0.11	0.12	0.15	0.03	1			
LAS_6	3.87	1.05	− 0.79	− 0.04	43.66	0.31	0.33	0.41	0.46	0.11	1		
LAS_7	3.22	1.11	− 0.33	− 0.84	37.68	0.43	0.35	0.45	0.51	0.09	0.49	1	
LAS_8	3.23	1.07	− 0.23	− 0.74	31.91	0.02	0.04	0.02	− 0.08	0.16	0.03	0.05	1

Note. LAS_1 … LAS_8: Loss Aversion Scale items. M: mean. SD: standard deviation. Sk: skewness. Ku: kurtosis. ^a^Polychoric correlations. AD: Anderson–Darling normality test.

Table 6 shows the association of LAS items for Sample 2’s external sociodemographic variables. The effect of all of them was very close to 0 for gender (M = 0.09), tobacco consumption in the past year (M = 0.03), age (M =  − 0.1), and the highest level of education attained or in progress (M =  − 0.04).

**Table 6:** Correlation of LAS items with external variables (sample 2, n = 766).

Variable	LAS_1	LAS_2	LAS_3	LAS_4	LAS_5	LAS_6	LAS_7	LAS_8
Gender	0.08	0.07	0.29	0.1	− 0.02	0.02	0.11	0.07
Tobacco	0.05	0.01	− 0.04	0.05	0.02	0.02	0.06	0.05
Age	− 0.06	− 0.13	− 0.16	− 0.13	− 0.02	− 0.19	− 0.12	− 0.01
Studies	− 0.02	− 0.06	− 0.03	− 0.04	− 0.04	− 0.04	− 0.08	0.02

Note. LAS_1 … LAS_8: Loss Aversion Scale items. None of the correlations are statistically significant.

### Internal structure

**Data quality.** The results of the Sample 2 analysis relating to overly consistent responses (G_+_ scores) placed threshold 72 as the cut-off point, eliminating 25 subjects from the sample. The cut-off point for inconsistent answers (O_+_ scores) was 30.5, which removed one subject from the sample. After the detection and elimination of subjects, the sample size was 766 for the subsequent analyses.

**Dimensionality.** The overall fit of the eight-item LAS was adequate in all the adjusted fit indices (CFI > 0.95, RMSEA < 0.05, SRMR < 0.05; Table 7). However, the local adjustment, assessed by the size of the factor loads, indicated two items below 0.50 (items 5 and 8). Item 5 was “Avoiding failure is less important to me than seeking success,” and item 8 stated, “The suffering that comes with losses can be fully offset by the pleasure that comes from gains.” Both were invertedly coded items and were removed. The measurement model was again adjusted with the remaining items. The new model showed better global and local fit (Table 7) and was statistically different from the first model: Δ*χ*^2^ = 15.83, *p* < 0.001 (Δdf = 2), ΔCFI = 0.011, ΔRMSEA = 0.012, Δ SRMR = 0.017. Due to the poor local fit (i.e., low factor loads) of the initial eight items, the authors accepted the six-item scale.

**Table 7:** Dimensionality of the LAS instrument.

	Unidimensional	Modified Unidimensional
LAS_1	0.616	0.614
LAS_2	0.582	0.58
LAS_3	0.667	0.662
LAS_4	0.754	0.765
LAS_5	0.155	–
LAS_6	0.610	0.608
LAS_7	0.692	0.692
LAS_8	0.026	–
Adjusted		
ULSMV *χ*^2^ (df)	52.157 (20)*	16.854 (9)*
CFI	0.985	0.996
RMSEA (CI 90%)	0.046 (0.031, 0.061)	0.034 (0, 0.058)
SRMR	0.044	0.032

Note: LAS_1 … LAS_8: Loss Aversion Scale items. ULSMV: unweighted least square mean variance. df: degrees of freedom. CFI: comparative fit index. RMSEA: root mean square error of approximation. CI: Confidence interval. SRMR: standardized root mean squared residual.

*p < 0.001.

**Equivalence between groups.** Table 8 reveals slight variations in magnitude across all compared groups during the invariance assessment. The exception was observed in the invariance of intercepts within the gender and age groups. Here, the ΔCFI exceeded |0.01| and the ΔRMSEA surpassed 0.10, indicating a lack of invariance at the scalar level. To probe the repercussions of intercept non-invariance, we computed the d_MACS_ indices.

**Table 8:** Invariance of the LAS instrument: gender, age, and consumption tobacco groups.

	ULSMV *χ*^2^ (df)	CFI	RMSEA (95% CI)	SRMR	Difference (Δ)		
					ΔCFI	ΔRMSEA	ΔSRMR
Gender					–	–	–
Configuration	24.856 (18)*	0.997	0.032 (0, 0.059)	0.038	–	–	–
Thresholds	40.614 (24)*	0.992	0.043 (0.018, 0.065)	0.038	− 0.005	0.011 (0.018, 0.006)	0
Factor loads	48.860 (29)*	0.991	0.042 (0.02, 0.062)	0.043	− 0.001	− 0.001 (0.002, − 0.003)	0.005
Intercepts	122.812 (34)*	0.958	0.083 (0.067, 0.099)	0.045	− 0.033	0.041 (0.047, 0.037)	0.002
Age					–	–	–
Configuration	23.910 (18)*	0.997	0.029 (0, 0.058)	0.038	–	–	–
Thresholds	30.928 (24)*	0.997	0.027 (0, 0.053)	0.038	00.00	− 0.002 (0, − 0.005)	.000
Factor loads	40.733 (29)*	0.994	0.033 (0, 0.054)	0.043	− 0.003	0.006 (0, 0.001)	.005
Intercepts	71.825 (34)*	.982	0.054 (0.036, 0.071)	0.043	− 0.012	0.021 (0.036, 0.017)	0.00
Tobacco					–	–	–
Configuration	22.809 (18)*	0.998	0.026 (0, 0.056)	0.038	–	–	–
Thresholds	46.701 (24)*	0.989	0.05 (0.028, 0.071)	0.038	− 0.009	0.024 (0.028, 0.015)	.00
Factor loads	51.121 (29)*	0.99	0.045 (0.023, 0.064)	0.041	0.001	− 0.005 (− 0.005, − 0.007)	0.003
Intercepts	63.768 (34)*	0.989	0.048 (0.029, 0.066)	0.042	− 0.001	0.003 (0.006, 0.002)	0.001

Note: ULSMV: unweighted least square mean variance. df: degrees of freedom. CFI: comparative fit index. RMSEA: root mean square error of approximation. CI: confidence interval. SRMR: standardized root mean squared residual.

*p < 0.001.

For the gender group, the d_MACS_ values suggested expected standardized differences in item responses due to non-equivalence, with M = 0.228 (Min = 0.082, Max = 0.560; Table 9). These values indicate trivial magnitudes (< 0.40) or low (< 0.60) levels. Similarly, the analysis extended to the age and tobacco use groups, maintaining the pattern of trivial or low levels. Moreover, negligible differences were observed in the expected observed scores. Therefore, this analysis concludes that the measurement invariance is maintained in all the groups analyzed, namely sex, age, and tobacco usage.

**Table 9:** Evaluation of non-equivalence: d_MACS_ effect size.

Item level	Gender		Age		Tobacco	
	d_MACS_	NEC	d_MACS_	NEC	d_MACS_	NEC
LAS_1	0.138	Trivial	0.247	Trivial	0.100	Trivial
LAS_2	0.149	Trivial	0.120	Trivial	0.016	Trivial
LAS_3	0.560	Low	0.258	Trivial	0.134	Trivial
LAS_4	0.242	Trivial	0.225	Trivial	0.118	Trivial
LAS_6	0.082	Trivial	0.417	Low	0.057	Trivial
LAS_7	0.198	Trivial	0.087	Trivial	0.102	Trivial
Total score level						
Expected mean differences	1.363		− 1.047		0.267	

Note: LAS_1 … LAS_7: Loss Aversion Scale items. d_MACS_: expected standardized difference. NEC: non-equivalence classification.

**Reliability.** Reliability using the omega coefficient was 0.724 (CI95% = 0.688, 0.758), and Cronbach’s alpha coefficient produced 0.703 (CI95% = 0.666, 0.736). The standard measurement error (*sme* = $SD/\sqrt {1 - reliability)}$ generated by the omega coefficient for the total sample expressed in z-units was 0.525; the *standard measurement error* generated by the alpha coefficient was 0.544.

### Association with other variables

Table 10 displays the outcomes of the LAS association with various variables, as calculated in Sample 2. Divergent exploration was examined by juxtaposing the LAS instrument with the Brief Sensation Seeking Scale and Glover-Nilsson Test and exploring its potential linkage to tobacco consumption over the past year. Furthermore, we scrutinized the correlation between LAS and Social Desirability Scale responses, employing the criteria outlined in the work of Lovakov and Agadullina^61^.

**Table 10:** LA score correlation with variables: total sample and gender group.

	Total sample^b^	Gender^b^		
		Man	Woman	Difference Z test
SS	− 0.133* (− 0.188**) ^c^	− 0.028^ns^	− 0.098^ ns^	0.024^ns^
TPD	− 0.015^ns^ (0.095^ns^)^c^	− 0.120^ns^	0.088^ns^	− 0.117^ns^
SD	0.332**	0.293**	0.354**	0.023^ns^
TC ^a^	− 0.050^ns^	0.017^ns^	− 0.082^ns^	0.024^ns^

Note: SS: sensation seeking. TPD: tobacco psychological dependence. SD: social desirability. TC: tobacco consumption (during the past year).

^a^Tobacco: Dichotomized variable (light consumption vs. habitual consumption).

^b^Pearson correlations from structural equation modelling (latent correlations).

^c^Biased correlations by social desirability score.

^ns^: *p* > 0.05; * *p* < 0.05; ** *p* < 0.01.

Anticipating a significant negative correlation between LAS and Brief Sensation Seeking Scale scores, albeit small, our analysis confirmed this expectation for the total sample (*r* =  − 0.133; *p* < 0.05). However, the anticipated Glover-Nilsson Test and tobacco use results were not fully realized, with insignificant correlations observed (*r* =  − 0.015 for Glover-Nilsson Test and *r* =  − 0.05 for tobacco consumption).

Conversely, a significantly moderately positive correlation emerged between Social Desirability Scale and LAS (*r* = 0.332; *p* < 0.01). This pattern persisted within both male (*r* = 0.293; *p* < 0.01) and female (*r* = 0.354; *p* < 0.01) subgroups.

Finally, we explored the influence of social desirability on the covariation between LA and other variables. The analysis revealed a negligible effect of social desirability on this relationship, evidenced by a minimal difference between coefficients (Fisher transformation difference = 0.05). A similar conclusion was drawn regarding the relationship between LA and psychological tobacco dependence (Fisher transformation difference = 0.11).

## Discussion

The overall objective of this study was to validate the LAS instrument^13^ within a Spanish population, aligning with recent recommendations from a comprehensive review on the construct of LA in the context of addictive behaviors^27^. To this end, we translated the instrument into Spanish, followed by rigorous assessments of content, internal structure, and validity, adhering to the Standards for Educational and Psychological Testing^43^. In contemporary psychological research, validation and reliability analyses are increasingly recognized as vital components of quantitative reporting^88^.

Addressing the specific objective 1, akin to findings in its original development^13^, Item 5 of the LAS instrument was deemed unsuitable for measuring LA and subsequently excluded. Substantive validity assessments and quantitative item analyses corroborated this outcome. Similarly, Item 8 exhibited analogous issues, warranting its exclusion based on substantive validity and item analysis findings. Notably, both items were formulated negatively, a characteristic that typically yields suboptimal results in Spanish-designed instruments^89^. Removing these items from the instrument notably enhanced its reliability and item-total correlation. Conversely, the remaining LAS items were deemed sufficiently clear and effective in measuring the intended construct^44,71,90,91^.

For the specific objective2, which focuses on validating the internal structure of the instrument, we conducted analyses on its dimensionality, measurement invariance, and reliability. The instrument’s authors originally proposed a one-dimensional structure^13^, which proved adequate upon assessment, particularly after removing items 5 and 8.

Since the unidimensional model was empirically robust and fitted with the instrument design, we found no rationale for testing multidimensional models of substantive factors. Additionally, we did not consider it necessary to include a method factor (e.g., random intercepts^92^) to capture idiosyncratic responses, as participants with potential response bias had already been excluded.

Consequently, a 6-item scale was deemed appropriate^73^. Measurement invariance analyses revealed consistent invariance between light and habitual tobacco use groups. However, there was a potential lack of invariance between men and women and between different age groups. Subsequent examination using the d_MACS_ index suggested that invariance between gender and age groups could be assumed, given the trivial differences^93^. Reliability was evaluated using two parameters, *ω* and *α*, in accordance with recent literature recommendations^94^. Our study demonstrated satisfactory reliability of LAS in a Spanish young adult sample, with both *α*^95^ and *ω*^96^ measures. However, caution is advised when considering clinical applications, as reliability levels are not optimal. Therefore, LAS is recommended primarily for general population studies. These findings support hypothesis 1, confirming that the LAS instrument is suitable for Spanish samples due to its promising validity and reliability.

Regarding the hypotheses linked to the specific objective 3, only hypothesis 3 was supported, indicating that individuals with high LA are less inclined to seek experiences that elicit new sensations. This finding aligns with recent research^54,55^. However, hypothesis 2 was unsupported, as the negative correlation was not statistically significant. Consequently, we couldn’t confirm that individuals with high LA are less likely to use tobacco or experience psychological dependence on it. This outcome could be attributed to an instrument measuring psychological dependence (the Glover-Nilsson Test)^58,62^ rather than the traditional instrument for assessing physical dependence, the Fagerström Test for Nicotine Dependence^97,98^. These results diverge from those reported in various articles, where consumers exhibit lower aversion to loss^48–50^. Furthermore, they contradict theoretical findings indicating that loss-averse individuals are more inclined to quit smoking^99^.

A surprising discovery emerged regarding hypothesis 4, where a moderately positive correlation was initially found, indicating that people with higher LA also have high social desirability tendencies. However, despite the significant positive regression observed between LA and social desirability, they exhibited a negligible covariance. Consequently, unlike the other variables, we cannot assert that the social desirability variable directly influences the LA variable, which displayed significant regression and covariance with social desirability. It is thus presumed that elevated levels of social desirability may impact responses to other questionnaires. Specifically, individuals with high social desirability tendencies while completing the questionnaire are likely also to score high in sensation seeking and low in psychological dependence on tobacco. This phenomenon appears intertwined with the contextual understanding of each variable within social dynamics.

The prevalence of high social desirability levels in social psychology studies, particularly those examining behaviors seeking social approval, underscores its significance^56,57^. Therefore, we contend that these are not merely biases but represent additional psychological variables worthy of consideration. Indeed, it has been proposed that certain variables should be correlated with social desirability, particularly those associated with behaviors enacted in public settings^100^.

### Limitations and future lines of research

In terms of study limitations, firstly, while the sample was meticulously selected according to specific criteria to facilitate analysis and generalization, its composition may have inadvertently marginalized diverse groups that could have provided valuable insights. For instance, the study’s sensitivity to cultural differences has been compromised, as individuals residing in Spain but originating from other countries were excluded, hindering the generalizability of the findings to Spanish-speaking contexts beyond Spain.

Secondly, the asymmetry observed in the age-divided sample groups introduces a potential for generating false positives or negatives in their invariance. However, our sample analysis revealed a small effect size, indicating minimal differences in sample distribution between the groups.

Continuing with the subject of age, another study limitation can be highlighted. As mentioned, age can be considered a moderator of LA, being more prevalent among older individuals^21^. Nonetheless, while this study does not show invariance concerning age, it is important to note that the sample is limited to a narrow demographic group consisting only of young adults (between 18 and 35).

Thirdly, there is a lack of literature confirming the variables correlated with LA as evidence of divergent validity. However, they can be considered additional exploratory analyses, which diminishes the validation power by missing this part.

Fourthly, a study limitation is the moderate effect size observed in the association between social desirability and LA. This relationship warrants further investigation as it may signify a significant variable of interest or reflect a desirability bias within the sample. Thus, we recommend that future research examine this aspect more thoroughly. Furthermore, regarding the lack of responses among a few categories, some variables were treated as continuous instead of categorial while performing the measurement invariance analyses. We used Multiple Linear Regression as the estimator.

Fifthly, another limitation of our study is the lack of a systematic comparison between Spanish-speaking and English-speaking samples to measure invariance across different populations, limiting our findings’ generalizability in a cross-cultural setting. Future research could include a detailed analysis to address this comparison.

Finally, it is important to remember the ongoing debate about the LA construct. Some studies have begun to question its validity, likely due to methodological flaws or misinterpretations of previous research^12,14–17^. Conversely, other studies support its existence, acknowledging that certain variables may modulate it^18–23^. Although the present study is theoretically aligned with the latter group, it did not include analyses directly to corroborate this approach.

Moving forward, we propose that future studies replicate the validation of this instrument in other Spanish-speaking countries, ensuring a broader representation of diverse populations. We advocate for sample selection strategies that enable a more nuanced exploration of gender variables. Additionally, investigating the correlation between gender and addictive behaviors is of paramount interest, given the inconsistencies in existing literature. It is imperative to examine this correlation in the context of addiction development, prevention, and treatment. Consequently, studies encompassing diverse samples will facilitate meaningful comparisons across different demographic groups. Studying generational differences among the Spanish population may be a good next step.

### Practical implications

This study holds significant practical implications, primarily confirming the accurate translation of the LAS instrument into Spanish and establishing its validity and reliability for assessing LA among young Spanish adults. This validation marks a pivotal advancement in LA research, particularly considering that the LAS is the first self-reporting instrument dedicated to this variable^13^. The study’s findings facilitate the seamless integration and utilization of the LAS in future investigations, enriching the landscape of LA research.

Furthermore, self-reporting measures, such as the LAS, are particularly noteworthy given recent literature indicating their superior reliability compared to measures derived from behavioral tasks analyzing the same variables^101^. Behavioral task measures analyze a person’s performance at a specific time, while self-reporting measures consider the habitual response. For this reason, self-reporting measures are deemed more suitable for studying individual differences.

Moreover, this study’s comprehensive reliability and validity assessment underscores the importance of transparency and replicability in future research endeavors. By adhering to standards of good practice advocated by various organizations^88,102^ and guidelines^103,104^, this work contributes to establishing robust methodologies and enhances the credibility of research outcomes within the scientific community.

## Conclusions

The findings of this study affirm the validity of the LAS instrument for application within Spanish-speaking populations. Through comprehensive analyses encompassing content, internal structure, and validity with other variables, our research underscores the suitability of this instrument for utilization across diverse population groups. Retaining six of its original eight items, the Spanish version of our study mirrored the one-dimensional structure observed in its English counterpart.

However, it is prudent to note that discrepancies emerged in the relationship between the LAS instrument and addictive behaviors within our population, deviating from expectations established in prior literature. Hence, a thorough review of this relationship is warranted. As the first validation study of the LAS instrument conducted in a language other than English, these results mark a significant initial stride towards its broader international adoption. Thus, these findings serve as a foundation for future research to refine and generalize the instrument’s applicability across diverse cultural and linguistic contexts. Finally, this study wishes to contribute to the current debate on the existence of the LA construct.

## Data Availability

The raw data supporting this article’s conclusions are available at 10.17605/OSF.IO/WCMVB. Correspondence and requests for any other materials should be addressed to Guillermo Chans.
